# Editorial: Optimizing Miscanthus for the Sustainable Bioeconomy: From Genes to Products

**DOI:** 10.3389/fpls.2018.00878

**Published:** 2018-06-25

**Authors:** Kerrie Farrar, Emily A. Heaton, Luisa M. Trindade

**Affiliations:** ^1^Institute of Biological, Environmental and Rural Sciences, Aberystwyth University, Aberystwyth, United Kingdom; ^2^Department of Agronomy, Iowa State University, Ames, IA, United States; ^3^Department of Plant Breeding, Wageningen University, Wageningen, Netherlands

**Keywords:** miscanthus, establishment and crop management, crop yield and phenology, biomass quality, Environmental impact, bioeconomy

In this Research Topic we report advances in fundamental and applied aspects of the perennial C4 bioenergy crop miscanthus (*Miscanthus* spp.) and its role in mitigating climate change as part of the bioeconomy. Miscanthus is a high yielding C4 perennial grass with great potential for sustainable biomass production in temperate climates, including Europe and North America as well as its native Asia. With high resource use efficiency and good biomass quality, miscanthus is a well-suited feedstock for a plethora of applications including bioenergy, biofuels and biomaterials (van der Weijde et al.).

Miscanthus offers a unique perspective within plant science: the challenge is to domesticate this novel crop for diverse environments and uses while simultaneously developing sustainable value chains to displace fossil fuels and contribute to climate change mitigation. Contributions to this Research Topic were offered from leading miscanthus researchers from different parts of the world. We accepted 16 articles from 95 authors, which have generated 21,161 views at March 26, 2018. Nine of the articles are the output of the European FP7 OPTIMISC project and describe multiple experiments investigating a common set of miscanthus genotypes in Europe and Asia. These papers are complemented by seven additional articles from global authors, providing a comprehensive analysis of the state of the art of miscanthus research and application.

Efficient **Establishment and Crop Management** are essential for this perennial crop that typically takes at least two growing seasons to reach commercial maturity, has an expected life span of over 10 annual commercial harvests, and receives minimal inputs after the planting phase. Within the OPTIMISC project, 15 miscanthus genotypes including *M. sinensis, M. sacchariflorus* and their hybrids, including the commercial clone *M*. × *giganteus* (*Mxg*), were planted in 6 locations across Europe and into Asia to determine the factors limiting to miscanthus production. Strong species x environment effects on biomass yield were observed, with the hybrids outperforming parental species in all locations except Turkey, where all types performed well. In the four Central/Southern locations, tested cultivars typically reached commercial yield in year two (Kalinina et al.). While temperature and rainfall were primary drivers of yield, soil fertility is also known to be important, especially as the crop matures.

An important consideration when establishing a non-native plant as a crop is the impact on native diversity. In the US, two miscanthus genotypes were introduced into different types of minimally managed landscapes and their invasive potential assessed over the following 5 years. Although there is concern that miscanthus has the potential to become invasive, only weak community impacts were observed. Furthermore, eradication can readily be achieved at early stages, emphasizing the requirement for early monitoring and management of this fast growing crop (West et al.).

Plant genotype and environment interact to impact **Crop Yield and Phenology**. Plant growth stages are coded systematically using systems such as the BBCH-scale which has been widely-adapted for a range of mono- and dicotyledonous plant species. Combining descriptions of close relatives with field observations Tejera and Heaton developed a simple BBCH scale for *Mxg* and related species, in which summary statistics can readily be estimated for a multi-stemmed plant. This enables improved comparisons between miscanthus and other species, and between miscanthus genotypes grown in different environments. The multilocation OPTIMISC trial comparing diverse miscanthus germplasm grown in six locations across Europe was used by Nunn et al. to determine the climatic limitations on biomass accumulation and inform the selection of trait combinations required to extend the boundaries of miscanthus cultivation. In panel analysis, biomass yield was assessed in 138 *Miscanthus sinensis* accessions collected in Southwest China and *M. sinensis* genotypes identified that matched or exceeded the biomass yield of *Mxg*. These corresponded generally to genotypes with high tiller numbers and plant height (Nie et al.). Together these studies provide insight into strategies for improving yield and resilience to stresses in miscanthus.

There have been conflicting reports on the effect of N fertilization on *Mxg* but Lee et al. determined that both biomass yield and the majority of yield component traits increased in value with N fertilization, and that the effect increased as the stands aged. In a separate experiment, N management was varied over two growing seasons, resulting in enhanced growth of above- and belowground tissues in the fertilized treatments with respect to the unfertilized control. Regular sampling of above and below ground organs was performed to observe the N dynamics throughout the growing season of the crop, and between growing years (Dierking et al.). Conversely, Jezowski et al. demonstrated the ability of *Mxg* to grow on degraded coal mine soils with or without high rates of sewage sludge and mineral fertilizer, indicating that *M*×*g* shows great growth potential for application on land that is unsuitable for other agricultural uses.

Similarly, the limitations imposed by soil or water salinity, an increasingly common abiotic stress globally, were assessed by collecting and assessing miscanthus from saline environments. Seventy genotypes of *M. sinensis, M. sacchariflorus* and interspecific hybrids, including the core genotypes from the OPTIMISC multilocation trial, were tested for growth under 150 mM saline in a hydroponic system. A range of responses was observed among the genotypes. Some of the relatively tolerant types accumulated little Na^+^ in the leaves, indicating an active Na^+^ exclusion mechanism while other genotypes showed reduced leaf growth, potentially demonstrating osmotic tolerance (Chen et al.). Taken together these studies provide insight into strategies for optimizing biomass production in miscanthus across a wide range of locations, encompassing climatic variation and land unsuitable for food production.

Three manuscripts describe the **Biomass Quality** of the 15 core OPTIMISC genotypes evaluated at six sites for three consecutive years for different end uses. Results highlight the great impact genotypic differences can have on the quality of biomass for bioenergy production, and interactions with the growth environment. In studies of biomass quality, this interaction was substantial and the authors concluded that it should be taken into account in breeding programs (Van der Weijde et al.). A subset of the OPTIMISC accessions, the five highest yielding genotypes in three locations, were further tested for combustion quality in different locations at different harvest times. he results showed that a delay in harvesting time improves combustion quality but at the expense of yield (Iqbal et al.). Kiesel et al. showed with another subset of the OPTIMISC accessions the effect of effect of the genotype, location and harvest date on energy yields of anaerobic digestion and combustion. In an independent experiment, Belmokhtar et al. studied the biomass yield and quality of five mature genotypes of *Mxg* and *M. sinensis* for three consecutive years (from years 3 to 5). They analyzed cell wall composition and saccharification efficiency using a miniaturized assay and revealed that more digestible genotypes contain a higher amount of hemicellulosic polysaccharides and lower amounts of lignin and cellulose.

For an energy, crop analysis of both the **Environmental impact and Bioeconomy** are critical as the energy produced must be commercially competitive with fossil fuels or other energy sources, yet more sustainable, particularly in terms of greenhouse gas emissions over the life cycle of the crop and conversion.

Ten field trials in different locations with various propagation and harvesting methods were compared for economic and environmental assessment of seed and rhizome propagated miscanthus in the UK. It was concluded that new hybrid seed propagation significantly reduces establishment costs with a breakeven yield of about half average UK typical yield for *Mxg*. Different harvesting and processing options are optimal for different end uses (Hastings et al.).

Understanding the environmental impact of miscanthus across a range of both value chains (e.g., heat and power, biomaterials) and hazard categories (e.g., human toxicity, marine toxicity) was advanced by the work of Wagner et al. who used results from the OPTIMISC multilocation trial to assess 36 growing site × pathway combinations. This is most holistic life-cycle assessment yet conducted on miscanthus. The place where miscanthus drove differences in environmental performance through biomass yield. While some miscanthus value chains showed large net environmental benefits, particularly to water toxicity and eutrophication, others could have net negative effects, particularly dependent on choice of reference scenarios and credits given for co-products. OPTIMISC accomplishments are summarized by Lewandowski et al. with many highlights, including data describing where and why miscanthus hybrids out yield *Mxg*; how to select and grow miscanthus for improved biomass quality, even under abiotic stress; advances in seed propagation and harvest; and end-user needs including life-cycle assessment.

This issue provides new insights in the understanding of miscanthus as a crop, including crop establishment and management, yield and crop phenology; the environmental impact of this crop and the suitability of biomass for biobased products.

## Author contributions

All authors listed have made a substantial, direct and intellectual contribution to the work, and approved it for publication.

### Conflict of interest statement

The authors declare that the research was conducted in the absence of any commercial or financial relationships that could be construed as a potential conflict of interest.

